# Enhancing the Physicochemical Attributes of Dough and Noodles through the Incorporation of *Bacillus vallismortis* Laccase

**DOI:** 10.3390/foods12224146

**Published:** 2023-11-16

**Authors:** Xiaoyu Zhu, Shijin Zhang, Luyao Bian, Juan Shen, Chong Zhang, Sivakumar Manickam, Yang Tao, Zhaoxin Lu

**Affiliations:** 1 College of Food Science and Technology, Nanjing Agricultural University, No. 1 Weigang, Nanjing 210095, China; zhuxiaoyu@njau.edu.cn (X.Z.); 2021808108@stu.njau.edu.cn (S.Z.); 2020208028@stu.njau.edu.cn (L.B.); 2016027@njau.edu.cn (J.S.); zhangchong@njau.edu.cn (C.Z.); yang.tao@njau.edu.cn (Y.T.); 2 Petroleum and Chemical Engineering, Faculty of Engineering, Universiti Teknologi Brunei, Bandar Seri Begawan BE1410, Brunei; manickam.sivakumar@utb.edu.bn

**Keywords:** *Bacillus vallismortis* laccase, cross-linking, dough, noodle, physicochemical properties

## Abstract

This investigation examined how the *Bacillus vallismortis* laccase (rBVL-MRL522) influenced the physicochemical characteristics, structural attributes, and functional capabilities of both dough and noodles. Incorporating rBVL-MRL522 (1 U/g) did not lead to a substantial change in the water absorption of wheat flour. However, the introduction of rBVL-MRL522 caused a significant elongation in the formation time of wheat flour dough, extending it by 88.9%, and also resulted in a 50% increase in the stabilization duration of wheat flour dough. Furthermore, adding rBVL-MRL522 led to a proportional rise in both the elastic and viscous moduli (G’’ of the dough, signifying that r-BVL (rBVL-MRL522) has a beneficial effect on the gluten strength of the dough. Integrating rBVL-MRL522 promoted the consolidation of the gluten-based cross-linked structure within the dough, decreasing the size of starch particles and, more evenly, the dispersion of these starch particles. In the noodle processing, adding rBVL-MRL522 at a rate of 1 U/g raised the L* value of the noodles by 2.34 units compared to the noodles prepared without the inclusion of rBVL-MRL522. Using a greater amount of rBVL-MRL522 (2 U/g) substantially increased the hardness of the noodles by 51.31%. Additionally, rBVL-MRL522 showed a noteworthy enhancement in the elasticity, cohesiveness, and chewiness of the noodles. In conclusion, rBVL-MRL522 promoted the cross-linking gluten, leading to a more extensive and condensed three-dimensional network structure in raw and cooked noodles. As a result, this study offers valuable insights into the environmentally friendly processing of dough and associated products.

## 1. Introduction

Today, products made from flour, especially those derived from wheat, play a significant role as fundamental dietary staples. They are a vital ingredient in the food industry and are widely used in supermarkets [1]. Compared to other flour types, wheat flour dominates the Chinese market, holding the largest share among flour-based products [2]. Nevertheless, differences in wheat types and cultivation conditions cause the medium or low gluten strength of the wheat flour produced. High gluten-strength flour is somewhat scarce, and the taste may not be optimal [3]. As consumers increasingly prioritize an enhanced quality of life, their expectations regarding the safety and quality standards of flour and flour-based products are rising [4]. As a result, there is a growing trend towards using safe flour enhancers to elevate flour quality. This has led to the development of custom-tailored flours designed for specific flour-based products, which has become a widespread commercial strategy [5]. This approach guarantees consistent and dependable quality for various specialized flours and improves the overall production efficiency of flour-based products.

Potassium bromate, a common chemical additive, is a potent gluten enhancer frequently employed in flour [6]. Initially regarded as safe, it gained extensive use in multiple food processing sectors, including the production of bread and pizza [7,8]. Potassium bromate exerts a gradual oxidative effect on the flour, enhancing the dough’s rheological characteristics and the cross-linking structure of gluten proteins. This, in turn, results in improved dough strength and elasticity, ultimately elevating the flavor and texture of baked flour-based products. Nonetheless, a troubling issue arises since potassium bromate leaves behind a residual amount in the finished baked products, presenting a carcinogenic risk even after the baking process [9]. Another frequently utilized chemical additive in flour is benzoyl peroxide, which acts as a bleaching agent [10]. Its bleaching process involves the oxidation and breakdown of unsaturated fat-soluble pigments in the flour, resulting in a loss of color [11]. Extended ingestion of benzoyl peroxide can lead to its buildup in the human body, potentially causing toxic effects and pathological changes in organs, such as the liver and kidneys, due to an excessive burden [12]. Hence, the generation of innovative enzyme-based flour quality enhancers to replace conventional additives like potassium bromate and benzoyl peroxide holds substantial importance in the processing of flour-based products. 

Laccase (EC 1.10.3.2), belonging to the multicopper oxidase family, is characterized by including four copper ions within its structure [13]. This enzyme exhibits remarkable versatility in substrates, enabling the catalysis of various compounds. These include natural macromolecules like lignin and humic acid and smaller molecules like phenolic and aromatic amine compounds [14]. An important feature of laccase is that it generates only water as a byproduct during oxidation [15]. Laccase plays a key role in the food industry by catalyzing the oxidative cross-linking of amino acids within peptides and proteins. This process results in the generation of a complex dough structure with improved gluten strength [16]. Fungal laccases have shown a higher catalytic efficiency in oxidizing patatin-enriched potato protein than potato protease inhibitors, even though the latter formed more efficient oxidative cross-linked products [17]. The cross-linking of potato proteins was achieved using either laccase alone or a laccase–ferulic acid system. In this context, ferulic acid enhanced the extent of cross-linking and the antioxidant activity in modified proteins [17]. Furthermore, as an oxidative-reductive enzyme, laccase is highly effective in catalyzing mycotoxin decomposition in food and animal feed, significantly reducing the safety risks associated with mycotoxin contamination [18]. Consequently, laccase can be regarded as an ideal flour enhancer for altering dough and flour-based products’ quality and sensory attributes.

In this study, “Yangmai” wheat flour was chosen as the primary raw material. The study aimed to assess how varying dosages of laccase from the *B. vallismortis* strain influenced dough formation and its properties, including tensile strength and extensibility. Additionally, the impact of rBVL-MRL522 on the elasticity, viscosity, and microstructure of the dough was examined. To investigate the effects of rBVL-MRL522 on flour-based products, noodles were taken as an example, and changes in whiteness, hardness, adhesiveness, chewiness, and elasticity were studied. Finally, a comparison was made between the microstructure of uncooked and cooked noodles after adding different laccase dosages. This study serves as a guide for applying laccase as a flour enhancer in the food industry. 

## 2. Materials and Methods

### 2.1. Materials and Chemicals

The wheat used in this study, specifically the “Yangmai” variety, was procured from Jiangsu Jintudi Seed Co., Ltd. (Nanjing, China) and stored at room temperature. The *Bacillus vallismortis* fmb-103 strain, which served as a source of the wild-type laccase gene, provided the genetic information necessary for laccase production. The genetic material was cloned into the Escherichia coli DH5α host for gene vector construction, and a subcloning expression host, Escherichia coli BL21 (DE3) pLysS, was employed for the expression of heterologous proteins. These strains used in this study were supplied by the Institute of Food Biotechnology at Nanjing Agricultural University. The cloning vector, pMD19-T, was obtained from TaKaRa Corporation’s subsidiary in Japan. The Escherichia coli expression vector, pET28a, was obtained from Novagen, a German-based company. Analytical grade potassium bromate and acetone were acquired from Shanghai Lingfeng Chemical Reagent Co., Ltd. (Shanghai, China). Isoamyl acetate (analytical grade), hydrochloric acid (analytical grade), and glutaraldehyde (analytical grade) were obtained from Nanjing Chemical Reagent Co., Ltd. (Nanjing, China). Analytical grade 2% osmium tetroxide was received from Shanghai Tungya Chemical Technology Development Co., Ltd. (Shanghai, China).

### 2.2. Preparation of rBVL-MRL522

The laccase gene was derived from *B. vallismortis* fmb-103, a gene previously cloned, identified, and stored in the laboratory. To obtain the laccase gene, it was amplified using the primers lac-F (forward): ATGACACTTGAAAAATTTGTGGATGC and lac-R (reverse): TTATTTATGGGGATCAGTTATATCCATCGGTC. The PCR process consisted of an initial denaturation step at 94 °C for 5 min, followed by 30 cycles of denaturation at 94 °C for 50 s, annealing at 55 °C for 1 min, extension at 72 °C for 30 s, and a final extension step at 72 °C for 10 min. The PCR amplification products were examined using gel electrophoresis, purified, and ligated into the pMD19-T vector, and their sequences were validated through sequencing conducted by Nanjing GenScript Corporation (Nanjing, China). 

The mutagenic laccase was generated by incorporating 0.05 U/μL of Taq DNA polymerase, 1 mM of Mg^2+^, 0.15 mM of dNTPs, and 0.1 mM of Mn^2+^. Following the DNA shuffling rearrangement, a laccase variant named MRL522 was produced, demonstrating an enzymatic activity of 8521 U/mg. The mutation sites in this variant were identified as T415G/R416G/T418N.

### 2.3. Preparation of Dough

The raw dough was prepared based on the Ji et al. method and procedure [19], in which a 4-α-glucan branching enzyme was added to prepare the dough. Here, the procedure is the same as their report, and the enzyme used is different. Wheat flour and water were mixed at a ratio of 5:3 (g:mL) and stirred for 30 min. Then, the mixture was rested in a dough maker (AB-P10C; ACA, Zhuhai, China) for 30 min at 30 °C. In wheat flour, 50 μg/g of potassium bromate or varying concentrations of rBVL-MRL522 (0.5, 1, or 2 U/g) were added to create the dough, and the native wheat flour, without any additives, served as the control group. The properties of the wheat flour were analyzed using a farinograph (Model:E-1; Brabender GmbH & Co. KG, Duisburg, Germany) with a 300 g dough mixing bowl, and the torque was set at 9.8 ± 0.2 mN·m/FU. The fast and slow mixing blade speeds were set at 63 ± 2 r/min and 31 ± 1 r/min, respectively.

### 2.4. Dynamic Rheological Properties of Dough

Dynamic rheological tests of the dough were carried out using a dynamic rheometer (MCR 302, Anton Paar, Graz, Austria) following the method of Song [20] and Tang [21]. A mold with a 4 cm diameter was employed, and the oscillation stress intensity was set at 30%. The scanning frequency ranged from 0.01 to 20 Hz, and the normal force range was between 0.1 and 10.0 N in order to obtain the linear viscoelasticity zone. These measurements were conducted at 25 °C. The water absorption of the wheat flour was determined using the method described by Okuda et al. [22] based on the farinograph curve, and an appropriate amount of water was added to achieve a dough consistency of (500 ± 20) BU at 25 °C. The dough was then allowed to rest and ferment for 1 h under the same temperature conditions. 

### 2.5. Microstructure of Dough

To observe the dough’s microstructure, we followed a method described by Guo et al., with the same procedure and different samples and instruments [23]. After adding 50 μg/g of potassium bromate or different concentrations of rBVL-MRL522 (0.5, 1, or 2 U/g), the wheat flour was thoroughly mixed using a farinograph equipped with a 300 g dough mixing bowl. Small dough samples, approximately 3–5 mm^3^, were then extracted and taken for testing. These dough samples were placed in a 2.5% glutaraldehyde solution, followed by three washes with a 0.1 M phosphate buffer. After fixation with 1% osmium tetroxide, the samples were subjected to a gradient dehydration process using ethanol, starting from 30% and increasing to 90%. Subsequently, ion sputter-coating was applied, and the samples were examined using a scanning electron microscope (SEM, Hitachi S-4800; Hitachi, Ltd., Tokyo, Japan). 

### 2.6. Wet Gluten Content

Precisely 20 g of wheat flour was measured and mixed with 50 μg/g of KBrO_3_, various concentrations of rBVL-MRL522 (0.5, 1, or 2 U/g), and water. These mixtures were allowed to ferment at 25 °C for 10 min as well as 60 min. Following fermentation, the doughs were carefully rinsed with deionized water, with kneading and rinsing repeated until the iodine solution in the washing water no longer exhibited color (indicating the complete removal of starch). Subsequently, the wet gluten content was determined by weighing the washed doughs [24]. 

### 2.7. Amino Acid Composition

The analysis of the free amino acids in the dough was conducted using a Shimadzu LC-2010A system (Shimadzu, Tokyo, Japan), based on Ijarotimi’s method with the same procedure and different instruments [25]. In summary, the washed doughs were frozen for 48 h to concentrate the protein content and then hydrolyzed with HCl at 110 °C for 24 h to release the free amino acids. The analytical column used was an Agilent 2622PH (4.6 × 60 mm I.D.). The flow rate was set at 0.40 mL/min, the column temperature was maintained at 57 °C, the reaction temperature was 135 °C, the detection wavelength was 570 nm, and the injection volume was 20 µL. HPLC-grade free amino acid standards (Asp, Ser, Glu, Gly, Ala, Cys, Val, Met, Ile, Leu, Tyr, Phe, Lys, NH3, His, Arg) with a purity greater than 99.9% were chosen as the standards for creating the calibration curves. The results have been reported in mg of each standard per liter.

### 2.8. Cooking of Noodles

The cooking of noodles followed the method described by Sui et al. [26]. A total of 200 g of wheat flour was measured, and rBVL-MRL522 was added at concentrations of 0.5 U/g, 1 U/g, and 2 U/g, respectively. Then, 70 mL of water at 30 °C was added, and the mixture was kneaded with a mixer for 5 min. Following this, the dough was allowed to ferment for 30 min. Afterwards, the dough was pressed six times to form noodles with a width of 3 mm and a length of 22 cm. Subsequently, 40 wet noodle samples were removed and placed in 600 mL of boiling water. Once cooked, the noodles were taken out of the boiling water and submerged in cold water for 10 s. Then, they were set on a sieve for 15 s before undergoing a comprehensive assessment.

### 2.9. Physicochemical Properties of Noodles

#### 2.9.1. Determination of Cooking Time

The noodle samples were boiled in boiling water for 4 min. At 30 s intervals, samples were withdrawn from the boiling water and promptly submerged in ice-cold water for 5 s. Subsequently, the noodles were flattened to determine where the white, hard line at the center of the noodle disappeared. This disappearance time was recorded as the optimal cooking time for the noodles [27].

#### 2.9.2. Determination of Noodle Water-Holding Capacity and Cooking Loss

In order to assess the water retention capacity of the cooked noodles, approximately 10 g of the cooked noodles were first weighed. They were then immersed in ice water at 0 °C for 5 s, after which they were removed and reweighed using a precision balance (in g) [28]. The water-holding capacity of the noodles was determined using Equation (1).

(1) $$\mathrm{Noodle}\;\mathrm{water}\;\mathrm{holding}\;\mathrm{capacity}\;\left(\%\right)=\frac{\mathrm{weight}\;\mathrm{of}\;\mathrm{cooked}\;\mathrm{noodles}\;\left(g\right)-\mathrm{weight}\;\mathrm{of}\;\mathrm{raw}\;\mathrm{noodles}\;\left(g\right)}{\mathrm{weight}\;\mathrm{of}\;\mathrm{raw}\;\mathrm{noodles}\;\left(g\right)}$$

A total of 100 mL of noodle soup was heated until it reached boiling point and was allowed to evaporate, resulting in a reduced volume of liquid. Subsequently, the remaining liquid was dried in a temperature-controlled oven at 105 °C until a constant weight was achieved. The cooking loss rate was determined using the following Equation (2).

(2) $$\mathrm{Loss}\;\mathrm{of}\;\mathrm{cooking}\left(\%\right)=\frac{5M}{G\times \left(1-W\right)}\;\times \;100\%$$

where *M* represents the weight of dried, solid material after drying, measured in grams (g); *W* denotes the moisture content of the noodles, expressed as a percentage (%); and *G* represents the weight of the noodles before the boiling process, measured in grams (g).

#### 2.9.3. Determination of Noodle Whiteness

The color distinction between the uncooked and cooked noodles was determined using a method based on the procedure of Zhu et al., with minor modifications [29]. Noodles were prepared following various formulations, including a blank control, 30 µg/g of potassium bromate, 0.5 U/g of rBVL-MRL522, 1 U/g of rBVL-MRL522, and 2 U/g of rBVL-MRL522. Subsequently, the L* (representing black to white), a* (indicating green to red), and b* (representing blue to yellow) values were measured using a colorimeter (CR-400, Konica Minolta, Tokyo, Japan).

#### 2.9.4. Texture of Noodles

The A/LKB model texture analyzer (Stable Micro System Ltd., Godalming, England) was utilized with specific settings, including a blade descent rate of 0.8 mm/s and a compression distance of 0.7 mm, to examine various textural characteristics of the noodles. These attributes encompass resilience, hardness, adhesiveness, chewiness, cohesiveness, and elasticity. For shear testing, the blade descent rate of the texture analyzer was adjusted to 0.17 mm/s, corresponding to 90% of the noodle thickness. During the analysis of the noodles, parameters such as maximum shear force, shear area, and shear time were measured.

#### 2.9.5. Microstructure of Raw and Cooked Noodles

Individual samples of raw and cooked noodles measuring around 3–5 mm^3^ in size were chosen for examination and imaging through a scanning electron microscope (SEM).

### 2.10. Statistical Analysis

The assessment of pertinent parameters of flour, gluten content, and amino acid content was carried out following a randomized design, including triplicate experiments and triplicate measurements, to calculate the mean value for each data point. A one-way analysis of variance (ANOVA) was conducted to assess the significance, and post hoc comparisons were performed using the Tukey multiple comparison test within SPSS (IBM, version 17.0). A significance level of *p* < 0.05 was used as the threshold for determining the statistical significance of the observed differences.

## 3. Results and Discussion

### 3.1. Effect of rBVL-MRL522 on Dough Farinograph Quality

The influence of KBrO_3_ and rBVL-MRL522 on the flour’s quality characteristics was evaluated using a farinograph, and the outcomes are displayed in Table 1. Compared to the control group, where KBrO_3_ was used, a 33% extension in dough development time and a 111% increase in dough stability time were observed. Additionally, the weakening effect on the flour decreased by 27.2%. Various concentrations of rBVL-MRL522 resulted in improvements in the dough stability time and a reduction in the weakening effect on the flour, thereby enhancing the quality of the flour. Both low and high concentrations of rBVL-MRL522 exhibited no impact on the dough development time. However, adding 1.0 U/g BVL-MRL522 notably enhanced the flour quality, resulting in increased water absorption, dough development time, stability time, and flour quality index compared to the control group using KBrO_3._ These results suggest that the enzyme rBVL-MRL522 offers stronger improvements in dough quality than KBrO_3_, demonstrating its potential as a promising dough strengthener. This implies that an appropriate laccase dosage can effectively enhance gluten strength in the dough. Nevertheless, excessive quantities of rBVL-MRL522 may decrease the flour’s powder quality index, which can significantly impact the overall processing quality. Typically, the dough-stretching ratio or tensile strength is a comprehensive indicator of dough processing performance [30]. This ratio encompasses two critical measures, dough extensibility and resistance to extension, and is utilized to evaluate flour processing quality [31]. A small ratio implies low resistance and high extensibility, making such dough susceptible to rapid softening and dispersion during fermentation and processing. Conversely, a large ratio indicates high resistance and strong elasticity but limited extensibility, potentially resulting in a firmer overall texture and reduced softness in the final baked products.

### 3.2. Effect of r-BVL on Dough Rheological Properties

**Figure 1:**
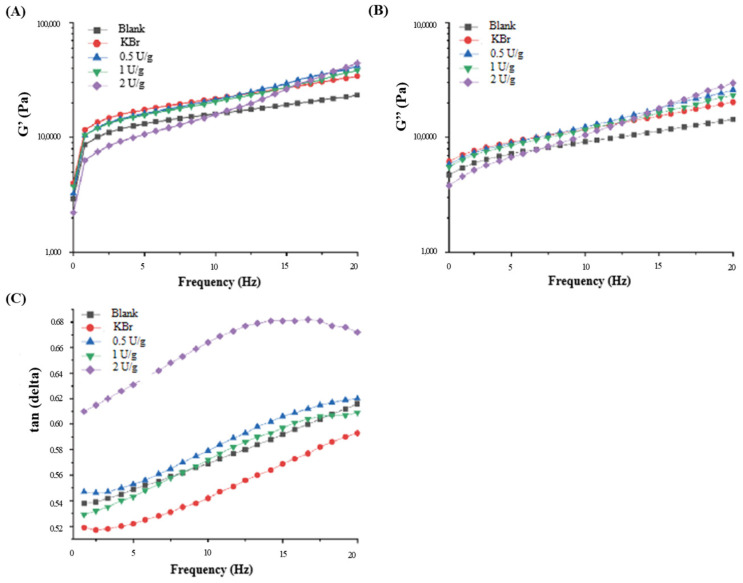
Effect of BVL-MRL522 on the storage modulus (**A**), loss modulus (**B**) and loss tangent value (**C**) of dough rheological properties (Note: KBrO_3_ group served as the negative control).

As illustrated in Figure 1, when 50 μg/g of KBrO_3_ or rBVL-MRL522 was introduced, the dough’s elastic and viscous moduli experienced corresponding increases. This observation suggests that rBVL-MRL522, acting as a dough strengthener, has a similar effect to the positive control, KBrO_3_. As the dosage of rBVL-MRL522 increased, the dough’s elastic and viscous moduli gradually increased. When the amount of rBVL-MRL522 added reached 2.0 U/g, the elastic modulus exhibited a 62.75% increase compared to the blank control, indicating that rBVL-MRL522 improved the dough’s ability to recover after deformation. The 59.35% increase in the viscous modulus suggests that rBVL-MRL522 enhanced the dough’s resistance to flow. The results for the loss tangent value (tan(delta)) indicated that as the addition of rBVL-MRL522 reached 0.5 and 1.0 U/g, tan(delta) gradually increased, signifying an increase in the proportion of polymers within the dough due to rBVL-MRL522. However, at a concentration of 2.0 U/g of rBVL-MRL522, tan(delta) first increased and then decreased, suggesting that excessive rBVL-MRL522 could affect the dough’s flexibility. Therefore, a careful addition of rBVL-MRL522 enhances the integrity of the dough’s gluten network, augments its flexibility, and significantly improves its resilience. These findings are consistent with the earlier flour quality tests, confirming that an optimal amount of rBVL-MRL522 enhances the dough’s strength. These results are consistent with the report of Manhivi et al. [32]. They observed the laccase-treated dough’s storage modulus (G’) and loss modulus (G″) significantly increased. The improvement in dough rheological properties may be attributed to the modification of proteins in the dough by disulphide bond formation, oxidative gelation of mucilage, as well as hetero-cross-linking between proteins and mucilage, which may strengthen the dough.

### 3.3. Effect of r-BVL on the Amino Acid Composition of Dough

#### 3.3.1. Effect of rBVL-MRL522 on Wet Gluten Content

The influence of rBVL-MRL522 on the moist gluten content in the dough is presented in Table 2. The results from the control group show that the fermentation time of the dough (10 min and 60 min) has a negligible impact on the moist gluten content. In contrast, KBrO_3_, acting as a slow oxidant, exhibits an insignificant strengthening effect on dough quality at a concentration of 50 μg/g when the dough mixing time is relatively short. In this case, the quality of the extracted moist gluten does not significantly differ from that of the control group. However, with the addition of rBVL-MRL522 at concentrations ranging from 0.5 to 2.0 U/g, the extracted moist gluten’s quality initially increases but then decreases. At a dosage of 1.0 U/g of the enzyme, the extracted gluten mass was 6.78 g, marking a 13.6% enhancement compared to the control group and a 12.8% improvement compared to the KBrO_3_ group. Furthermore, doughs to which rBVL-MRL522 was added after 60 min of incubation led to a higher extracted moist gluten than those incubated for 10 min. This implies that rBVL-MRL522 acts as a fast-acting oxidant, exerting oxidative effects on thiol groups during the dough mixing stage. The oxidative effect becomes more pronounced as the incubation time increases, resulting in more extracted moist gluten. Nonetheless, an excessive addition of rBVL-MRL522 can reduce the extractable moist gluten content in high-dough formulations. In summary, the findings suggest that adding an appropriate amount of rBVL-MRL522 can boost the extractable moist gluten content in the dough.

#### 3.3.2. Changes in Amino Acid Composition among Different Gluten Samples

The level of polar amino acids and disulfide bonds in wheat gluten proteins is a crucial indicator of dough strength [33]. Analyzing alterations in the amino acid composition of gluten samples following the introduction of rBVL-MRL522 necessitates evaluating dough strength shifts.

As shown in Table 3, the hydrolyzed amino acid content in gluten initially increased and then decreased within the range of 0.5–2.0 U/g with the addition of rBVL-MRL522. However, all values were significantly higher than those in the control group. Particularly, when 1.0 U/g rBVL-MRL522 was introduced, the total content of the hydrolyzed amino acids increased by 12.80% compared to the control group. The levels of polar amino acids, including histidine, arginine, lysine, aspartic acid, and glutamic acid, experienced an increase. Glutamic acid (Glu) demonstrated the most noteworthy change, exhibiting a 17.50% increase compared to the control group. This implies that rBVL-MRL522 augmented the protein content within the gluten. However, there was a 31.30% decrease in the content of cysteine (Cys), indicating a reduction in the number of cysteine residues in the gluten. This suggests that rBVL-MRL522 has a robust strengthening effect. On the other hand, rBVL-MRL522 increased the content of the essential aminos crucial for human health, such as threonine (Thr), valine (Val), isoleucine (Ile), and phenylalanine (Phe). These amino acids can serve as additives to enhance the nutritional value of wheat-based products.

### 3.4. Effect of r-BVL on Dough Microstructure

**Figure 2:**
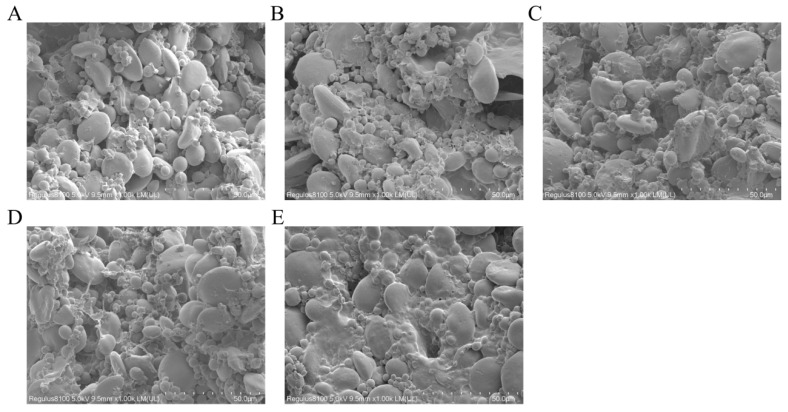
Comparison of microscopic analysis of doughs enhanced with different improvers. (**A**) control dough; (**B**) dough with 50 μg/g KBrO_3_; (**C**) dough with 0.5 U/g rBVL-MRL522 included; (**D**) dough with 1.0 U/g rBVL-MRL522 added; (**E**) dough with 2.0 U/g rBVL-MRL522 added.

As depicted in Figure 2, the outcomes of the SEM analysis of dough samples from various experimental groups showcased evident microstructural differences. Upon magnification to 1000 times, the dough from the blank control group displayed an absence of clearly defined gluten networks. Starch granules were readily visible, and the structure appeared loose and poorly cohesive with the granular arrangement. With the introduction of KBrO_3_, an improved gluten cross-linking effect was evident, leading to a greater encapsulation of starch granules by gluten proteins. Following this, with the incorporation of rBVL-MRL522, the network structure of gluten in the dough became progressively stronger as the enzyme dosage increased, leading to a higher encapsulation of starch granules. This led to intricate, interlinked gluten structures, forming a complex three-dimensional spatial network. However, when 2.0 U/g of rBVL-MRL522 was added, the dough’s surface appeared smoother and more cohesive than the other groups. This suggests that excess rBVL-MRL522 may disrupt the gluten network and increase the dough’s extensibility. The production of phenoxyl radicals, facilitated by the oxidation of ferulic acid within the flour by rBVL-MRL522, fosters cross-linking among feruloyl arabinoxylans. This, in turn, causes the dough’s ability to resist stretching. Additionally, laccase catalyzes the formation of disulfide bonds by oxidizing thiol groups in gluten proteins. This process contributes to the overall enhancement of mechanical strength and stability of the dough [34].

### 3.5. Effect of r-BVL on Cooking Characteristics of Noodles

To improve the gluten strength of noodles, in addition to using high-gluten flour, other agents have been suggested [35]. For example, introducing glutamine transaminase into noodles can substantially increase gluten strength, resulting in enhanced chewiness and cookability [36]. Furthermore, incorporating alkali water into flour can significantly bolster gluten formation [37]. In the manufacturing of hand-pulled noodles, alkaline water is an essential component. The heightened cross-linking of gluten proteins promotes the creation of a network structure in noodles, which, in turn, minimizes the release of substances into the cooking liquid when boiling. Consequently, this leads to a decrease in noodle loss, prevents clumping, and enhances the texture of the noodles. Nevertheless, it is worth noting that relatively limited studies are available on enhancing noodle quality through biocatalyst utilization. Throughout the cooking process, starch granules in noodles undergo gelatinization, absorbing water, which can result in the partial dissolution or suspension of starch in the cooking liquid. As shown in Table 4, the incorporation of rBVL-MRL522 led to a 3.9% increase in the rate of water absorption by the noodles, along with a 4.9% decrease in the loss rate. Moreover, there is a minor extension in the optimal cooking time. 

This phenomenon can be attributed to the catalytic effect of rBVL-MRL522 in generating hydrogen peroxides from polyunsaturated fatty acids, promoting the formation of disulfide bonds within gluten proteins. These disulfide bonds intensify the cross-linking between gluten protein molecules, improving gluten strength. Establishing and reinforcing the gluten protein network structure contributes to increased water absorption by the dough, reduced starch dissolution, and a decreased loss rate. However, an excessive amount of rBVL-MRL522 can lead to an overly strong gluten strength, reduced water absorbency, an extended cooking time, and an elevated loss rate for the noodles.

**Figure 3:**
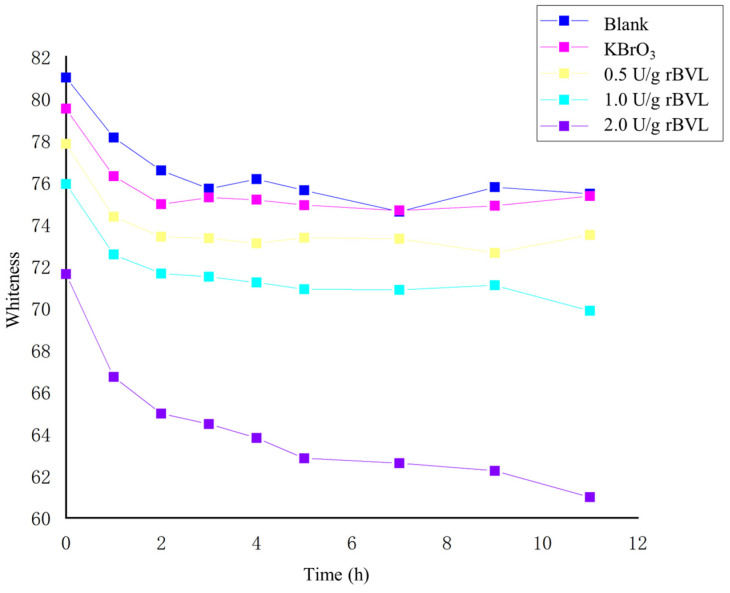
The whiteness of noodle samples prepared with the addition of KBrO_3_ or rBVL.

**Figure 4:**
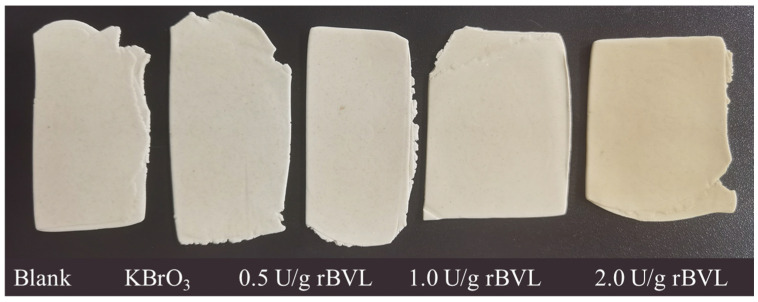
Different noodle samples prepared with the addition of KBrO_3_ or rBVL.

The whiteness of the noodles in the different treatment groups was assessed using a colorimeter, and the effect of different levels of rBVL-MRL522 addition on noodle whiteness is presented in Figure 3. The results reveal that, except for the 2.0 U/g addition, the impact of the rBVL-MRL522 addition on the whiteness of the noodles is not significant. Excessive addition of rBVL-MRL522, such as at the 2.0 U/g level, causes the noodles to exhibit a yellowish hue, diminishing their whiteness. This color change may be associated with the inherent color characteristics of the enzyme preparation used in the laboratory. Figure 4 displays the different noodle samples obtained from the experiment, aligning with the color difference findings illustrated in Figure 3.

### 3.6. Effect of r-BVL on the Texture of Raw and Cooked Noodles

Hardness, adhesiveness, and chewiness were chosen as criteria to evaluate the firmness, hardness, and elasticity of the noodles, along with various other attributes (Table 5). The findings reveal that the inclusion of 1.0 U/g of rBVL-MRL522 led to notable improvements in the noodles’ hardness, adhesiveness, and chewiness. Specifically, compared to the control group, the addition of 1.0 U/g of rBVL-MRL522 resulted in an increase of 8.5% in the raw noodle’s hardness, an increase of 8.05% in adhesiveness, and an increase of 12.49% in chewiness. The enhancement in the quality of cooked noodles resulting from rBVL-MRL522 was notably superior to that observed in raw noodles. Following the addition of 1.0 U/g of rBVL-MRL522, the cooked noodles exhibited a substantial 22.87% increase in hardness compared to the control group, along with a 13.65% boost in adhesiveness and a 17.08% increase in chewiness. These results imply that rBVL-MRL522 can greatly improve the firmness and elasticity of noodles, particularly when they are cooked. This result is consistent with the report of Gui et al. [38], where the addition of laccase contributed to a higher hardness, cohesiveness, and chewiness of the protein gel.

The maximum shear force is intricately linked to the texture of noodles. With the incorporation of rBVL-MRL522, there was an evident increase in the shear force of the noodles. Specifically, when the addition of rBVL-MRL522 was 1.0 U/g, the maximum shear force of raw noodles led to a substantial increase of 34.60% in comparison to the control group, signifying that rBVL-MRL522 enhances the cookability of noodles, rendering them chewier. However, when the addition of rBVL-MRL522 was increased to 2.0 U/g, the strong gluten effect disappeared, aligning with the outcomes of the previous experiment. This underscores the importance of using the right quantity of rBVL-MRL522 to enhance noodles’ cookability. Furthermore, KBrO_3_, a potent gluten enhancer, notably amplified the maximum shear force of cooked noodles. Specifically, when the addition of rBVL-MRL522 reached 1.0 U/g, the maximum shear force of the noodles led to a significant increase of 31.80% compared to the control group. It exceeded that of the KBrO_3_ control group. This demonstrates that an appropriate quantity of rBVL-MRL522 can enhance the noodles’ ability to resist deformation.

### 3.7. Effect of r-BVL on the Microstructure of Raw and Cooked Noodles

**Figure 5:**
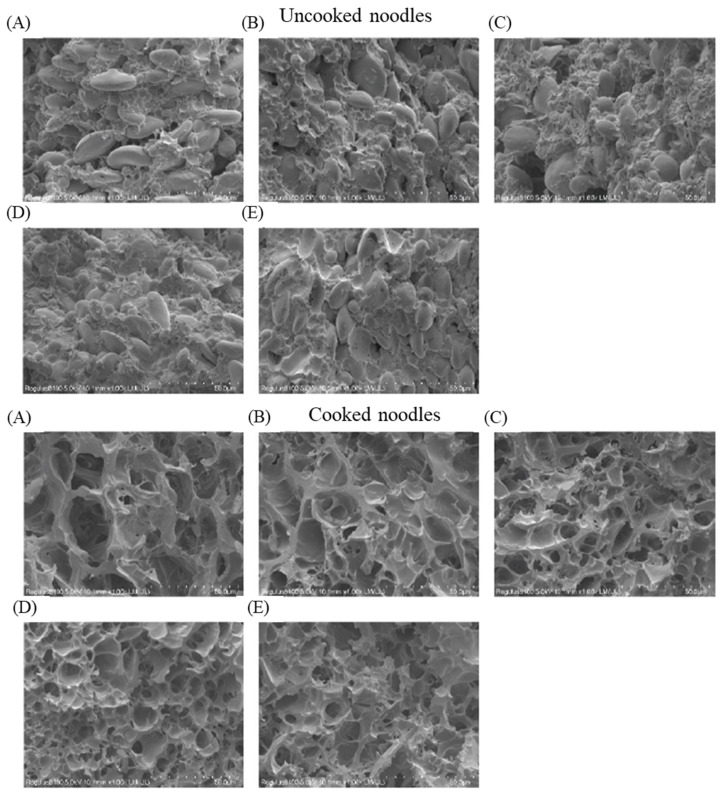
Comparative analysis of the microscopic structure in uncooked and cooked noodles. (**A**) control dough; (**B**) dough with 50 μg/g KBrO_3_ addition; (**C**) dough with 0.5 U/g rBVL-MRL522 incorporation; (**D**) dough with 1.0 U/g rBVL-MRL522 inclusion; (**E**) dough with 2.0 U/g rBVL-MRL522 addition.

The influence of rBVL-MRL522 on the microstructure of raw and cooked noodles was examined using SEM, and the findings are illustrated in Figure 5. Incorporating rBVL-MRL522 or KBrO_3_ intensified the gluten cross-linking effect within the noodles, resulting in a more pronounced fibrous network structure of gluten. Furthermore, it led to a more uniform distribution of starch granules, with gluten tightly enveloping them.

Moreover, it is important to note that starch has the potential to undergo gelatinization when subjected to boiling, leading to a more prominent gluten structure in cooked noodles as opposed to their raw counterparts [39]. As proteins experience denaturation due to heat, a stable network structure is established, and the starch granules are completely hydrolyzed within the cross-section of the noodles, forming cavities [40]. In the case of cooked noodles supplemented with rBVL-MRL522, these cavities within the starch granules were noticeably smaller and displayed a uniform distribution. The internal structure displayed denser and more porous features, as illustrated in Figure 5. This suggests that rBVL-MRL522 enhances the gluten’s tensile strength within the noodles, consequently enhancing the overall quality of the noodles. Based on this, adding rBVL-MRL522 contributes to improving the noodles’ firmness, elasticity, and overall mouthfeel. Furthermore, the internal gluten structure of the noodles forms a more intricate and complex fibrous network, ultimately elevating the noodles’ overall quality of the noodles.

## 4. Conclusions

The impact of incorporating rBVL-MRL522 on the quality of both dough and noodles was examined. In investigating its strengthening effect on the gluten networks in the dough, rBVL-MRL522 was observed to significantly enhance the formation of wheat flour dough, resulting in an extended stability period for the dough. When rBVL-MRL522 was added at a rate of 2 U/g, it increased both the elastic modulus (G′) and viscous modulus (G″) of the dough by 62.75% and 59.35%, respectively, in comparison to the negative control group. Additionally, the introduction of rBVL-MRL522 achieved a higher interconnection of gluten networks, translating to increased resistance to dough stretching. The compound also demonstrated enhanced capabilities in encapsulation, resulting in a more even dispersion of starch particles within the dough. It is worth noting that the inclusion of rBVL-MRL522 led to a decrease in cysteine (Cys) content in the dough, coupled with elevated levels of glutamic acid (Glu) and aspartic acid (Asp). Furthermore, an increase in the contents of threonine (Thr), valine (Val), isoleucine (Ile), and phenylalanine (Phe) was observed. In enhancing the quality of noodle products, incorporating rBVL-MRL522 improved the hardness and whiteness of the cooked noodles. Moreover, rBVL-MRL522 was found to facilitate the interconnection of gluten networks, thereby enhancing the firmness, adhesiveness, chewiness, and elasticity of the noodles. These findings collectively suggest the potential of rBVL-MRL522 as a promising, safe, and residue-free bio-additive for enhancing the quality of flour-based products.

## Figures and Tables

**Table 1:** Effects of rBVL-MRL522 on dough farinograph quality.

Group	Water Holding Capacity (%)	Formation Time (min)	Duration of Stability (min)	Degree of Attenuation (FU)	Flour quality Index
Blank	60.7 ± 0.2 ^b^	0.9 ± 0.1 ^b^	1.8 ± 0.2 ^d^	125.4 ± 5.0 ^a^	18.3 ± 2.1 ^b^
50 μg/g KBrO_3_	60.9 ± 0.1 ^b^	1.2 ± 0.1 ^a^	3.8 ± 0.3 ^a^	91.0 ± 3.1 ^c^	30.5 ± 3.2 ^a^
0.5 U/g BVL-MRL522	60.8 ± 0.2 ^b^	0.9 ± 0.1 ^b^	2.7 ± 0.2 ^b^	103.2 ± 7.3 ^b^	27.2 ± 3.2 ^a^
1.0 U/g BVL-MRL522	61.2 ± 0.2 ^a^	1.7 ± 0.2 ^a^	2.4 ± 0.2 ^c^	88.4 ± 4.4 ^c^	31.4 ± 2.2 ^a^
2.0 U/g BVL-MRL522	60.9 ± 0.2 ^b^	0.9 ± 0.1 ^b^	2.2 ± 0.1 ^c^	108.1 ± 2.2 ^b^	27.2 ± 4.3 ^a^

Note: Different lowercase letters indicate statistical significance (*p* < 0.05) within each column.

**Table 2:** Effect of rBVL-MRL522 on the wet gluten extraction ratio.

Group	10 min		60 min	
	Mass of Wet Gluten (g)	Extraction Yield (%)	Mass of Wet Gluten (g)	Extraction Yield (%)
Blank	5.9 ± 0.2 ^aA^	29.6 ± 0.4 ^b^	6.0 ± 0.1 ^aA^	29.8 ± 0.2 ^b^
KBrO_3_ 50 μg/g	6.0 ± 0.2 ^aA^	29.8 ± 0.3 ^b^	6.0 ± 0.2 ^aA^	30.0 ± 0.3 ^b^
rBVL-MRL522 0.5 U/g	6.1 ± 0.2 ^abA^	30.7 ± 0.5 ^a^	6.6 ± 0.1 ^bB^	32.5 ± 0.4 ^a^
rBVL-MRL522 1.0 U/g	6.2 ± 0.1 ^bA^	31.2 ± 0.7 ^a^	6.8 ± 0.1 ^cB^	32.8 ± 0.2 ^a^
rBVL-MRL522 2.0 U/g	6.0 ± 0.1 ^aA^	30.2 ± 0.6 ^a^	6.5 ± 0.3 ^bcB^	31.9 ± 0.3 ^a^

Note: Different lowercase letters indicate statistical significance (*p* < 0.05) within each column. Different uppercase letters indicate statistical significance (*p* < 0.05) within each row.

**Table 3:** Effect of rBVL-MRL522 and KBrO_3_ on the amino acid composition (mg/g dry gluten) in gluten.

Types of Amino Acids	Control	KBrO_3_	rBVL-MRL522		
			0.5 U/g	1.0 U/g	2.0 U/g
Asp	26.67 ± 0.22 ^d^	30.92 ± 0.24 ^a^	30.99 ± 0.26 ^a^	30.24 ± 0.18 ^b^	28.48 ± 0.17 ^c^
Thr *	22.03 ± 0.32 ^c^	25.53 ± 0.37 ^a^	25.64 ± 0.43 ^a^	25.20 ± 0.27 ^a^	23.64 ± 0.21 ^b^
Ser	37.23 ± 0.27 ^c^	47.99 ± 0.66 ^a^	48.32 ± 0.38 ^a^	47.90 ± 0.39 ^a^	44.41 ± 0.41 ^b^
Glu	289.09 ± 1.44 ^c^	336.32 ± 2.02 ^a^	338.57 ± 1.13 ^a^	339.67 ± 2.05 ^a^	313.55 ± 1.42 ^b^
Gly	27.07 ± 0.52 ^d^	30.15 ± 0.22 ^b^	30.39 ± 0.31 ^ab^	30.79 ± 0.21 ^a^	28.31 ± 0.27 ^c^
Ala	22.46 ± 0.37 ^c^	25.40 ± 0.17 ^a^	25.61 ± 0.32 ^a^	25.58 ± 0.26 ^a^	23.80 ± 0.31 ^b^
Cys	29.20 ± 0.41 ^a^	20.65 ± 0.24 ^c^	21.10 ± 0.42 ^bc^	21.48 ± 0.20 ^b^	20.07 ± 0.26 ^c^
Val *	35.34 ± 0.35 ^c^	38.35 ± 0.32 ^a^	38.53 ± 0.19 ^a^	38.48 ± 0.18 ^a^	36.08 ± 0.22 ^b^
Met *	14.16 ± 0.33 ^b^	15.81 ± 0.41 ^a^	15.95 ± 0.31 ^a^	15.54 ± 0.37 ^a^	14.76 ± 0.25 ^b^
Ile *	31.34 ± 0.25 ^c^	34.05 ± 0.22 ^a^	34.12 ± 0.20 ^a^	34.18 ± 0.26 ^a^	32.12 ± 0.31 ^b^
Leu *	59.39 ± 0.21 ^d^	65.66 ± 0.19 ^b^	66.13 ± 0.37 ^a^	66.27 ± 0.42 ^a^	61.68 ± 0.37 ^c^
Tyr	26.36 ± 0.19 ^d^	29.30 ± 0.20 ^b^	30.16 ± 0.28 ^a^	30.24 ± 0.26 ^a^	27.05 ± 0.28 ^c^
Phe *	44.31 ± 0.27 ^b^	47.31 ± 0.31 ^a^	47.76 ± 0.27 ^a^	47.99 ± 0.31 ^a^	44.45 ± 0.33 ^b^
Lys *	16.87 ± 0.33 ^b^	17.68 ± 0.11 ^a^	17.80 ± 0.21 ^a^	17.91 ± 0.19 ^a^	16.81 ± 0.24 ^b^
His	17.63 ± 0.35 ^b^	19.12 ± 0.19 ^a^	19.19 ± 0.10 ^a^	19.32 ± 0.26 ^a^	17.97 ± 0.16 ^b^
Arg	31.50 ± 0.31 ^c^	34.37 ± 0.37 ^a^	34.70 ± 0.17 ^a^	34.35 ± 0.38 ^a^	32.27 ± 0.30 ^b^
Pro	93.59 ± 0.42 ^c^	104.03 ± 0.63 ^a^	104.84 ± 0.52 ^a^	105.23 ± 0.57 ^a^	97.41 ± 0.39 ^b^
Total	824.23 ± 1.45 ^d^	922.63 ± 2.15 ^b^	929.78 ± 1.09 ^a^	930.37 ± 1.92 ^a^	862.86 ± 1.33 ^c^

Note: * indicates essential amino acids, and *n* = 3. Note: Different lowercase letters indicate statistical significance (*p* < 0.05) within each column.

**Table 4:** Effect of adding rBVL-MRL522 on the cooking characteristics of noodles.

Group	Moisture Content (%)	Water Holding Capacity (%)	Loss Rate (%)	Optimum Cooking Time (%)
Blank	31.50 ± 0.41 ^a^	111.60 ± 2.1 ^a^	25.00 ± 2.11 ^b^	5.20 ± 0.35 ^ac^
KBrO_3_ 50 μg/g	31.50 ± 0.31 ^a^	114.50 ± 2.1 ^ab^	21.90 ± 1.90 ^ab^	5.80 ± 0.43 ^ab^
0.5 U/g rBVL-MRL522	32.00 ± 0.36 ^a^	115.60 ± 2.4 ^b^	22.90 ± 2.45 ^ab^	5.70 ± 0.67 ^ab^
1.0 U/g rBVL-MRL522	33.10 ± 0.40 ^b^	120.50 ± 1.9 ^c^	20.10 ± 1.89 ^a^	6.00 ± 0.36 ^b^
2.0 U/g rBVL-MRL522	32.00 ± 0.29 ^a^	115.70 ± 1.8 ^b^	32.70 ± 2.13 ^c^	4.90 ± 0.29 ^c^

Note: Different lowercase letters indicate statistical significance (*p* < 0.05) within each column.

**Table 5:** Effect of rBVL-MRL522 on noodle texture parameters.

Group		Hardness	Gumminess	Chewiness	Resilience	Cohesiveness	Springiness
Raw noodle	Sample 1	93.76 ± 0.21 ^c^	25.85 ± 0.35 ^b^	13.85 ± 0.29 ^b^	0.21 ± 0.09 ^b^	0.25 ± 0.07 ^b^	0.54 ± 0.04 ^c^
	Sample 2	95.99 ± 0.23 ^b^	24.83 ± 0.31 ^c^	13.91 ± 0.41 ^b^	0.21 ± 0.06 ^b^	0.26 ± 0.06 ^b^	0.56 ± 0.03 ^c^
	Sample 3	95.54 ± 0.18 ^b^	24.52 ± 0.27 ^c^	14.14 ± 0.22 ^b^	0.22 ± 0.05 ^b^	0.26 ± 0.08 ^b^	0.55 ± 0.05 ^c^
	Sample 4	101.73 ± 0.27 ^a^	27.93 ± 0.33 ^a^	15.58 ± 0.46 ^a^	0.23 ± 0.07 ^b^	0.28 ± 0.06 ^b^	0.57 ± 0.03 ^c^
	Sample 5	93.46 ± 0.22 ^c^	25.55 ± 0.24 ^b^	13.96 ± 0.39 ^b^	0.22 ± 0.08 ^b^	0.25 ± 0.09 ^b^	0.53 ± 0.02 ^c^
Cooked noodle	Sample 1	10.80 ± 0.12 ^g^	8.06 ± 0.21 ^f^	9.66 ± 0.29 ^e^	0.49 ± 0.07 ^a^	0.70 ± 0.05 ^a^	1.20 ± 0.03 ^ab^
	Sample 2	11.79 ± 0.17 ^f^	8.50 ± 0.37 ^e^	10.45 ± 0.21 ^d^	0.49 ± 0.06 ^a^	0.72 ± 0.04 ^a^	1.23 ± 0.01 ^ab^
	Sample 3	12.73 ± 0.19 ^e^	9.04 ± 0.28 ^d^	11.29 ± 0.36 ^c^	0.50 ± 0.05 ^a^	0.71 ± 0.07 ^a^	1.24 ± 0.04 ^ab^
	Sample 4	13.27 ± 0.16 ^d^	9.16 ± 0.25 ^d^	11.31 ± 0.29 ^c^	0.52 ± 0.09 ^a^	0.75 ± 0.04 ^a^	1.25 ± 0.02 ^a^
	Sample 5	11.84 ± 0.13 ^f^	8.52 ± 0.21 ^e^	10.16 ± 0.26 ^d^	0.48 ± 0.11 ^a^	0.71 ± 0.08 ^a^	1.19 ± 0.03 ^b^

Note: Different lowercase letters indicate statistical significance (*p* < 0.05) within each column. Samples 1–5 represent blank, 50 μg/g KBrO_3_, 0.5 U/g rBVL-MRL522, 1.0 U/g rBVL-MRL522, and 2.0 U/g rBVL-MRL522, respectively.

## Data Availability

Data is contained within the article.
